# What are our options for mortality data collection and how can they provide HIV-specific information?

**DOI:** 10.4102/jphia.v16i1.733

**Published:** 2025-05-27

**Authors:** Carlie L. Sulpizio, Zaena Tessema, Diane Morof, Andrew Boyd, Elfriede Agyemang, Martha Knuth, Danielle Fernandez, Monita Patel, Hammad Ali

**Affiliations:** 1 Division of Global HIV and Tuberculosis, United States (US) Centers for Disease Control and Prevention, Atlanta, GA, United States of America

**Keywords:** HIV/AIDS, mortality, surveillance, data collection, civil Registration, vital statistics

## Abstract

**Background:**

Mortality data are critical for understanding changes in population health, detecting and monitoring diseases, guiding public health responses and evaluating interventions like human immunodeficiency viruses (HIV) prevention and treatment programmes. However, in low- and middle-income countries (LMICs), comprehensive mortality data are often hindered by a high incidence of deaths occurring outside healthcare facilities and the lack of robust data systems, creating a significant knowledge gap.

**Aim:**

This article presents methodologies for collecting mortality data, particularly in LMICs, to provide accurate and reliable information on overall and cause-specific mortality, including HIV-related deaths.

**Setting:**

This study explore methods that may be useful in LMICs, where mortality data systems are often ad-hoc, sub-national and incomplete.

**Method:**

Available methods were examined for collecting mortality data and report on the strengths, weaknesses and resource considerations for each method.

**Results:**

The analysis shows that while Civil Registration and Vital Statistics is the gold standard for mortality data collection, its implementation is challenging because of differing priorities of stakeholders, infrastructural and legal barriers. Alternative methods may provide valuable data but may have limitations in coverage and resource allocation. Integrating these methods can enhance understanding of mortality data, including for HIV-related deaths.

**Conclusion:**

Implementing a combination of mortality data-collection methods could address gaps in mortality data in LMICs. Tailoring interventions based on these data may improve health outcomes and support HIV epidemic control efforts.

**Contribution:**

This study could be used as a resource to ministries of health, national and international public health organisations, researchers and funding bodies as it can assist countries in selecting the mortality surveillance strategy that best fits their HIV epidemic, and available infrastructure and financial resources.

## Background

Mortality data, when viewed comprehensively, can uncover changes in population health status over time and can be obtained through various methods to help understand these changes, including the ability to detect the initial cases of infectious diseases, monitor preventable diseases, implement public health response, raise awareness and provide insights to prevent further deaths. Mortality data help identify the causes and circumstances of death and thus provide essential information for an evidence-based response to changes in population health status.^1,2,3^ Cause of death (CoD) is the official determination of the condition leading to death. Cause of death is currently classified using the International Statistical of Diseases and Related Health Problems (ICD) codes.^4^ International Statistical Classification of Diseases and Related Health Problems is the base used for mortality coding and classification for diseases, disorders, injuries and other health-related conditions with multiple uses such as on death certificates or use by healthcare centres for billing. Currently, routinely available mortality data are limited in most low- and middle-income countries (LMICs) since most deaths occur at home in these settings.^5^ Where implemented, the mortality data systems are ad-hoc or sub-national, and the CoD data are incomplete or unreliable.^6^ Data quality is low for 85 countries capturing CoD data; and 74 countries, predominately in sub-Saharan Africa, do not capture mortality data in a routine surveillance system.^7^

Mortality data can also assist in assessing the impact of human immunodeficiency viruses (HIV) prevention and antiretroviral therapy (ART) treatment programmes. Trend data on HIV-related deaths can help HIV programmes determine if deaths among people living with HIV are increasing, decreasing or remaining stable. This information, along with CoD information, can help countries design or improve their existing HIV programmes and improve patient outcomes, such as viral suppression.

Implementing mortality systems is also important as they, in combination with information on new infections, can assist in improving our understanding of HIV epidemic control.^7^ One of the definitions of epidemic control is the point at which the total number of new HIV infections falls below the total number of deaths from all causes among HIV-infected individuals while both exhibit a declining trend (additional conditions for using this metric include a minimum ART uptake of 70% and a falling trend of new HIV infections). As countries get closer to HIV epidemic control, it is critical to understand the remaining burden and find creative solutions to HIV prevention, care and treatment. The lack of accurate mortality data can potentially hinder countries documenting HIV epidemic control. Currently, mortality data among people living with HIV, especially in countries in sub-Saharan Africa, come from either reporting death from the next of kin to the healthcare facility (which is limited) or through the follow-up of patients that miss their clinical appointments. Many deaths are missed in both these cases, and CoD information is generally not available. Therefore, mortality estimates for many LMICs, including most countries in sub-Saharan Africa, are based on modelling (e.g. Spectrum model for HIV, which is used to generate annual HIV estimates in 170 countries).^8^

There is a dearth of published papers that provide information on methods to collect mortality data. Here we aim to describe various methods, listed further in the text, that can be utilised to gather mortality data.^9^ These methods provide mortality data, including overall and cause-specific mortality rates and trends, and CoD information including HIV as the CoD. Strengths and weaknesses, along with resource and implementation considerations, of each method are described in Table 1.

**TABLE 1:** Strengths, weaknesses, availability of cause of death and representativeness of different mortality data-collection methods.

Type of system	Strengths	Weaknesses	Cause of death	Representativeness	Implementation considerations
CRVS	Gold standard for mortality data; can produce ongoing cause-specific mortality trends comparable over time and geographic area	CoD data reported to CRVS are frequently not available, inconsistent or the quality of data is unreliable; is resource-intensive and only works well with complete or near-complete coverage	CoD available if medical certification of death is entered into the CRVS	National coverage	Depends on scale of implementation (national/sentinel) *where CRVS exists in some form:* SWOT analysis; stakeholder meetings; data extraction; data analysis; dissemination
Burial systems	Captures all deaths, including community deaths that are brought to burial sites; can be supplemented with VA	Deaths not brought to registered burial sites are missed; VA-related weaknesses if VA is conducted	All CoD may be available if VA is conducted	Depends on the coverage	Training; human resources; development of tools and registers; monitoring; data entry; data analysis; dissemination
Household surveys with mortality modules	Wide variety of survey methods; can be added as a supplement to an existing survey	Requires skilled and trained interviewers and adequate financial resources; may require additional resources if VA is included in a second survey phase	All CoD may be available if VA is conducted	May be representative based on sampling approach	Training; human resources; development of surveys; field/data collection; data management; data entry; data analysis; dissemination
Hospital data on cause-specific mortality	Uses existing data; can be combined with community-based mortality data sources to improve representativeness	Does not capture community deaths if used alone; absence of ICD-trained professionals may contribute to limited or inaccurate coding	CoD available through medical records	May be representative based on coverage of facilities	Training; human resources; development of tools; data entry; monitoring; data analysis; dissemination
LTFU tracking	Uses existing HIV programme data; can support efforts to re-link people living with HIV LTFU back into care and treatment services	Only captures mortality among people living with HIV on treatment; needs to be supplemented with VA to assign a CoD if not available through medical records	CoD only available if medically certified CoD is provided by next of kin	May be representative of all people living with HIV on treatment if all sites are included	Training; human resources; development of tools; communication; transport; data entry; data analysis; dissemination
HIV CS	Provides unique information on HIV mortality and can help define epidemic control; consists of routine reporting of longitudinal individual-level data; facilitates HIV programmes to monitor and address gaps	Most low- and middle-income countries rarely collect mortality data, including CoD; lack of mortality data hinders the measurement of epidemic control	CoD only available if the country collects mortality data or links from other data systems	Depends on the coverage	Training and capacity building; human resources; development of data tools; communication and coordination; transport and logistics; data collection; data integration; data analysis; dissemination
Autopsy	Most reliable method of CoD determination	Resource-intensive; time-consuming; conducted by trained medical professionals	Conducted to determine CoD	Depends on the coverage	Training (including verbal autopsy training); human resources; development of tools; field/data collection; ethical considerations; data entry; data analysis; dissemination; quality assurance
VA	Can be used when deaths are not assigned a medical certificate of CoD, as in the cases of poorly functioning healthcare systems or deaths that occur in the community	Requires skilled and trained interviewers, data entry and database support and either physician-coded or automated assignment of CoD; can be inaccurate for certain causes (e.g., TB)^36^	All CoD available from VA	Depends on the coverage	Training (including CoD training); human resource; development of data tools; field/data collection; data entry; data analysis; dissemination
SAAVY	Can be representative of larger geographic or administrative area if statistical sampling scheme is used; requires fewer resources compared to full CRVS	Same as VA	All CoD available from VA	May be representative based on sampling approach	Same as CRVS and Verbal Autopsy
MIA/MITS	Sensitive in determining CoD compared to full post-mortem autopsies for infectious causes; less costly and time-consuming and more acceptable than full autopsy	Requires that pathology and laboratory infrastructure and personnel be in place (or be developed); less useful for non-infectious causes compared to full autopsy	CoD available based on the tests conducted on cadaver samples	Depends on the coverage	Training; human resources; equipment; development of tools; field/sample collection; sample transport; laboratory testing; data entry; data analysis; dissemination
HIV biomarker surveillance in mortuaries	Only blood or oral fluid/saliva is collected, making specimen collection easier than MIA; can provide information on underlying (undiagnosed) HIV disease, ART status, and viral suppression	May require trained staff that can collect blood from the cadaver; if conducted in mortuaries, it may be limited to urban settings where the majority of mortuaries exist	Only available if extracted through medical records	Not representative	Training; human resources; equipment; development of tools; field/sample collection; sample transport; laboratory testing; data entry; data analysis; dissemination

CRVS, Civil Registration and Vital Statistics; CoD, cause of death; SWOT, Strengths, weaknesses, opportunities and threats; VA, verbal autopsy; ICD, International Statistical Classification of Diseases and Related Health Problems; LTFU, loss to follow-up; HIV, human immunodeficiency viruses; CS, case surveillance; TB, tuberculosis; SAAVY, Sample Vital Registration with verbal autopsy; MIA, minimally invasive autopsy; MITS, Minimally Invasive Tissue Sampling; ART, antiretroviral therapy.

## Civil registration and vital statistics

Civil registration is defined as the continuous, permanent, compulsory and universal recording of vital events (births and deaths) pertaining to the population, as provided through decrees or regulations in accordance with the legal requirements in a country.^10^ Establishing a legal requirement obliges all citizens to abide by said requirement so that burials cannot take place if the death is not registered.^7^ Civil Registration and Vital Statistics (CRVS) systems – when fully functional, with national coverage, and including CoD – are the gold standard for assessing mortality rates and trends in a country. A national CRVS system includes registration of all deaths, irrespective of location, along with medically certified CoD. Data captured by the CRVS system can generate continuous information on health indicators, help understand the prevalence, distribution and causes of mortality, as well as the health inequities affecting mortality to inform programmatic and policy decisions. Many LMICs record vital events including births and deaths, but they may not cover the entire population, generate data on a continuing basis, capture accurate CoD especially for those dying outside of health facilities or be linked electronically.^6,11^ Therefore, these countries cannot compile data for accurate, detailed and ongoing analyses. Cause of death is a critical part of CRVS and should ideally come from medical certification. However, many countries fail to capture accurate CoD because of a lack of medical certification of deaths which can be because of multiple factors including lack of training for deaths occurring at health facilities and no clinical oversight for deaths occurring in the community. A high-functioning national CRVS system remains the gold standard for national mortality surveillance; however, achieving such a system is complicated by legal mandates, infrastructure and differing priorities of stakeholders.^6^

For HIV, CRVS systems can help assess the impact of HIV programmes and treatment on mortality in the total population. In many countries without a CRVS system, many deaths that occur outside of health facilities are not counted, thus leaving gaps in mortality data.^7,12^ Therefore, maintaining comprehensive CRVS includes necessary infrastructure, community participation and medical certification of CoD. Countries could conduct national assessments of their CRVS system and develop strategies to address gaps in mortality data.

## Burial systems surveillance

Mortality surveillance can be conducted with engagement of staff at burial sites. Each death brought to the burial site is registered in the surveillance system and basic demographic information is collected as part of registration. Cause of death is recorded if the cadaver has been brought from a healthcare facility or a mortuary (in case an autopsy is conducted at the mortuary). For cadavers brought from the community, verbal autopsy (VA) may be conducted by trained personnel to determine the CoD. Burial systems surveillance can also be used to calculate mortality trends using local population size.^13,14^

## Household surveys with mortality modules

In countries without medical certification of CoD or CRVS, mortality questions added to household surveys may provide a framework to understand the leading CoD in a population.^7,15^ Although VA modules are the most commonly used method to collect information on CoD in household surveys, supplemental methods may include a follow-up survey or a post-census survey.^7^ Follow-up surveys typically take place within a year of a death identified through the initial survey.^16^ Some countries may include additional modules such as the social autopsy, which identifies situations that may have affected mortality risk. Results from additional questionnaires help inform future survey methodology and strategies to improve mortality data capture.^17^ Post-census surveys are similar to follow-up surveys with the key difference being that in post-census surveys, deaths are identified through a census, and a sample of households with recorded deaths are followed up with VA interviews.

## Hospital data on cause-specific mortality

Routinely collected hospital data are a main source of cause-specific mortality statistics. Hospital data on cause-specific mortality are guided by the following HIV mortality principles: (1) recording deaths individually by patient age, sex, CoD and location of the hospital, (2) recording deaths in line with ICD principles when more than one cause is reported, (3) maintaining confidentiality and (4) sharing local mortality statistics with certifying clinicians.^7^ Clinical staff are trained on proper reporting of diagnosis and CoD data. Decision tables may also help staff accurately identify complicated underlying CoD.^18^ Hospitals can consider creating a local ICD coding index categorising the most frequent diagnoses and including comorbidities and sequences of events leading to death.^19,20,21^

## Loss to follow-up tracking

Loss to follow-up (LTFU) tracking is a method specific to HIV treatment programmes, although it can be utilised for other CoD including tuberculosis (TB). Many deaths among people living with HIV which occur outside the healthcare system go unreported and are incorrectly deemed ‘lost to follow-up’ in HIV programme data; this can lead to incorrect mortality estimates among people living with HIV receiving HIV services.^22,23^ A few methods have been proposed to correct mortality estimates.^23^ In settings with active death registration systems, notified deaths can be linked to the patient’s ART records, allowing documentation of the outcome of the LTFU tracking.^6,24^ In settings without adequate civil registration records or with incomplete, low-quality CoD data, tracing of individuals LTFU can be used to determine the current status and reason(s) for interruptions of treatment by surveillance and/or clinic staff via phone calls, home visits or social networks.^23,25^ If the death of an individual who has been LTFU is confirmed, the civil record can be updated by submitting a death report form to the national death registry, and mortality estimates can be adjusted accordingly. In addition to helping improve HIV mortality estimates, LTFU tracking is crucial to people living with HIV monitoring and improving their clinical outcomes.

## Human immunodeficiency viruses case surveillance

For HIV-specific mortality surveillance, mortality status, along with CoD, can be included as a part of existing HIV case surveillance (CS) systems. Human immunodeficiency viruses case surveillance consists of routine reporting of longitudinal individual-level data on all people living with HIV in a population from the point of diagnosis and through the cascade of sentinel events including treatment, viral suppression and death. Routine CS facilitates HIV programmes to monitor and address gaps in HIV service delivery and health outcomes, including mortality, among people living with HIV. However, CS systems are currently limited in scale in most LMICs, rarely collect mortality data, including CoD and face similar challenges as LTFU. When CS systems are limited or do not collect mortality data systematically, data from other mortality systems can be linked to CS to inform mortality data or bridge gaps in mortality data in CS. Designing an HIV CS system depends on the country context. Availability of well-established patient management systems facilitates reporting of data from sentinel events, including death, in the design of CS systems. Mortality data in HIV CS can assist in understanding the factors that contribute to mortality in HIV-positive individuals.^11,26^

## Autopsy

An autopsy is a surgical examination of a body to determine the cause and manner of death or to evaluate any disease or injury. Although histological examination related to autopsy is the most reliable method of determining CoD, an autopsy is not commonly used especially in LMICs, as autopsies are time-consuming and are conducted by trained medical professionals.^27^ In many LMICs, autopsies are only performed for medico-legal cases or upon request of the family of the deceased.

## Verbal autopsy

Verbal autopsy is an interview-based method, used to determine the probable CoD, in which an interview is conducted with the next of kin or caregivers after a death occurs outside of a healthcare setting.^28^ The interview, normally conducted by a trained field worker, is guided by a structured survey and gathers information on signs and symptoms related to illnesses exhibited by the deceased prior to death.^29^ For HIV, the VA questionnaire asks if the deceased ever had a positive HIV test or was diagnosed with AIDS, among other questions. Data are then analysed by physicians or automated computerised models to assign probable CoD. Many studies have been conducted to validate various VA approaches in the absence of CRVS, ranging from physician review of VA to automated and data-derived algorithms. For comparability, there are standaridsed VA guidance and data-collection instruments.^28,30,31,32^ Resources for training, evaluation and careful ethical consideration when discussing death in various cultures and populations are important in creation and maintenance of a successful VA system.^33^

## Sample vital registration with verbal autopsy

Sample vital registration with verbal autopsy (SAAVY), although not a long-term replacement for CRVS, can provide birth, death and CoD data needed for policy and programmes. Sample vital registration with verbal autopsy is a combination of CRVS and VA where vital event registration is completed in a sample of surveillance areas. Verbal autopsy is used to determine the CoD for deaths in a sample of surveillance sites, selected through probability sampling (as opposed to national coverage for CRVS). Following the selection of sites, a census is conducted at each site to gather baseline information about the population. Key informants in each site routinely monitor and report vital events and a VA is conducted for every death. The key difference between SAVVY and CRVS is that CRVS systems should have national coverage and be routine and ongoing, whereas SAVVY is done in a sample of surveillance sites.

## Biomarker-based sentinel surveillance

Full autopsies are considered the gold standard for determining CoD; however, they are cumbersome, require expertise and resources and have low acceptability. Autopsies, and associated testing, can help identify HIV in undiagnosed individuals and determine HIV-related CoD. In place of full autopsies, biomarker-based sentinel surveillance can provide trends in cause-specific mortality and can be conducted in two ways.

### Minimally invasive autopsy/minimally invasive tissue sampling

Minimally invasive autopsy (MIA)/minimally invasive tissue sampling (MITS) is a histopathological and biomarker testing of tissue and non-tissue samples, including body fluids, collected from a cadaver through minimally invasive techniques. It may also include imaging of cadaver. Minimally invasive autopsy/MITS sampling consists of collecting samples using biopsy needles from cadavers in a facility or from community deaths brought into a facility post-mortem. Samples can be tested for biomarkers including HIV, antiretrovirals (ARV), HIV drug resistance, TB, malaria, influenza, severe acute respiratory syndrome coronavirus 2 (SARS CoV-2) and opportunistic and other infections. Moreover, multi-pathogen PCR detection cards can be used for additional pathogen identification, and MIA/MITS can help identify other CoD including cancers. A multi-country study showed that MIAs/MITS are highly acceptable as a tool to determine CoD.^34^ Samples should be collected as soon as possible and ideally within 24 h.^35^ Minimally invasive autopsy/MITS is more invasive as it collects multiple tissue and non-tissue specimen from the cadaver, whereas only blood or oral fluid is collected through biomarker surveillance.

### Human immunodeficiency viruses biomarker sentinel surveillance in mortuaries

Human immunodeficiency viruses (HIV) biomarker sentinel surveillance can be conducted in mortuaries through collection of blood or oral fluid and/or saliva from cadavers. All cadavers admitted to a mortuary are included in the sentinel surveillance system. Blood is collected from the heart cavity of the cadaver through a transthoracic needle, and oral fluid and/or saliva is collected from the mouth of the cadaver. The sample is then sent to a laboratory for HIV testing and, in the case of blood collection, for other biomarker testing, including viral load, ARVs and HIV drug resistance. Blood samples may additionally be tested for other non-HIV biomarkers. Information collected through the biomarker testing can be supplemented by medical records if the death occurred at a health facility; however, in the event of a community death, this information may be unavailable.

## Conclusion

While LMICs work to implement CRVS, other methods can supplement or fill gaps in mortality data. The following factors could be considered when deciding which method is most feasible in the local context: methods of data collection, monitoring and storage; availability of electronic data systems versus reliance on paper-based systems; availability of laboratory infrastructure and skills; availability of human resources and technical skills of staff and availability of funding. Countries could also assess their existing infrastructure, resource availability and local mortality data need to determine the most feasible methods for implementation. In addition, cultural sensitivities around HIV and death in different communities should be considered, as acceptability of each method may vary across cultures. In LMICs, prioritising low-cost methods such as verbal autopsies or burial surveillance, combined with targeted training for local staff could help to address immediate mortality data gaps while laying the groundwork for more long-term CRVS development. Future research could potentially explore CRVS implementation challenges in LMICs, as a deeper understanding of these barriers may support more targeted and effective advocacy efforts while strengthening this gold standard system.

All these methods provide information on mortality generally, which includes mortality among people living with HIV and HIV as the CoD. Loss to follow-up tracking and HIV case surveillance are specific to mortality among people living with HIV and may provide information on other CoD among people living with HIV. With increasing numbers of people living with HIV in treatment and care, HIV-specific mortality data may be helpful to monitor and address gaps in epidemic control. This need has underscored the lack of consistent and routinely available mortality data. Although many of the existing mortality systems in LMICs are ad-hoc, and thus data from these systems often cannot be systematically and routinely used to inform national mortality estimates, they are still an important effort, as they provide extremely useful data that are not available otherwise. In the long run, mortality data-collection systems could be implemented in an ongoing or periodic manner so mortality data, including CoD data, are available over time. Data from these mortality methods can help prevent deaths by tailoring interventions to a particular region, disease or population.
